# Parent-led neonatal pain management—a narrative review and update of research and practices

**DOI:** 10.3389/fpain.2024.1375868

**Published:** 2024-04-16

**Authors:** Alexandra Ullsten, Marsha Campbell-Yeo, Mats Eriksson

**Affiliations:** ^1^ Center for Clinical Research and Education, Region Värmland, Karlstad, Sweden; ^2^ Faculty of Medicine and Health, School of Health Sciences, Örebro University, Örebro, Sweden; ^3^ School of Nursing, Faculty of Health, Dalhousie University, Halifax, NS, Canada; ^4^ MOM-LINC Lab, IWK Health Halifax, Halifax, NS, Canada

**Keywords:** parent, newborn, infant, neonate, procedural pain, pain management, family-centered

## Abstract

**Introduction:**

Research related to parent-led neonatal pain management is increasing, as is the clinical implementation. Skin-to-skin contact, breastfeeding and parents' vocalizations are examples of pain reducing methods that give parents an opportunity to protect their infant from harm while alleviating their anxiety and developing their parenting skills.

**Methods:**

In this paper we will provide a narrative review and describe the current research about parent-led neonatal pain management. Based on this we will discuss clinical challenges, implementation strategies and implications for future research.

**Results:**

Parents express great readiness to embrace opportunities to increase their self-efficacy in their ability to address infant pain. Parent-led pain-reducing methods are effective, feasible, cost-effective, culturally sensitive, and can be individualized and tailored to both the parent's and infant's needs. Both barriers and facilitators of parent-led pain care have been studied in research highlighting structural, organizational, educational, and intra- and interpersonal aspects. For example, health care professionals' attitudes and beliefs on parent-led methods, and their concern that parental presence during a procedure increases staff anxiety. On the other hand, the presence of a local pain champion whose duty is to facilitate the adoption of pain control measures and actively promote parent-professional collaboration, is crucial for culture change in neonatal pain management and nurses have a key role in this change. The knowledge-to-practice gap in parent-led management of infants' procedure-related pain highlight the need for broader educational applications and collaborative professional, parental and research initiatives to facilitate practice change.

**Conclusion:**

Parent-led neonatal pain management is more than simply a humane and compassionate thing to do. The inclusion of parent-led pain care has been scientifically proven to be one of the most effective ways to reduce pain associated with repeated painful procedures in early life and parents report a desire to participate. Focus on enablers across interprofessional, organizational and structural levels and implementation of recommended pediatric pain guidelines can support the provision of optimal evidence-based family-centered neonatal pain management.

## Introduction

Although family-centred care was introduced globally in paediatric and neonatal care in the 1990s (1, 2), it was not until the 21st century that many neonatal intensive care units (NICU) started to implement family-centred care welcoming both parents and possibly also siblings in the units, actively involving the parents in the infant's daily care round the clock. Still, accepting parents as active partners in neonatal pain management*,* is an even more recent and currently ongoing concept across NICUs and healthcare globally. The combined negative effect of repeated pain exposure and maternal separation is a strong stressor with adverse long-term effects (3), which further motivates having the parents at the unit and involving them in the pain management of their infant.

What we do know from previous research is that there is a research-to-practice gap among the NICU team when supporting parent-led pain-reducing methods and a combination of these, for example combined breastfeeding, skin-to-skin contact (SSC) and the parent's live singing during painful procedures (4). Moreover, while it is essential to include parents as active members in the NICUs interprofessional healthcare team since interprofessional collaboration contributes to improved procedural pain management in neonates, few NICUs have fully embraced parent inclusion (5). To what extent and consistency health care professionals promote, support, facilitate and systematically implement parent-led pain management globally, is yet to be ascertained. Nevertheless, engaging parents as key partners which includes promoting parent-led pain-relief and parental involvement in infants' pain assessment, is aligned with best evidence informed infant pain care and should be advanced.

This paper aims to provide a narrative review that describes the current best evidence for parent-led neonatal pain management, as well as address clinical challenges, implementation strategies and implications for future research.

## Review

### Parent-led pain assessment and management

Parents can play an active role in both assessment and management of neonatal pain (6). Parents who are able to be present with their infant at the hospital can identify signs of pain and discomfort (7–9). However, parents may underestimate their infant's pain, compared to the assessment of health care professionals (10, 11). As such, it is important that parents receive education and support from health care professionals to enable them to perform a valid and reliable pain assessment (12).

Several reviews have demonstrated that parents can be part of the pain management for their infant (4, 13–15) and that this is safe and effective (15). Some of the evaluated methods are almost solely performed by the mother (breastfeeding) or parent (SSC), whereas there are a number of methods that can be performed by the parents, other family members or health care professionals (e.g., facilitated tucking, providing non-nutritive sucking or swaddling) (16). There is moderate to high certainty evidence that skin-to-skin contact, and breastfeeding are effective pain-reducing methods, whereas other interventions should be seen as adjuvant or working best in combination with other (6).

#### Skin-to-skin contact

SSC, or Kangaroo Care (KC), means placing the baby dressed only in a diaper, and maybe a hat to avoid heat-loss, on the bare chest of the parent. Usually, an upright prone position is used, but side-lying, e.g., during breastfeeding, is also common. There are special tube tops or cloth slings that are used to support maintaining the KC-position over prolonged time.

SSC is probably the most studied parent-led pain relieving method for procedural pain. SSC has been demonstrated to significantly reduce pain intensity scores as measured using validated composite biobehavioral pain indicators (17), with an effect that sustains over repeated episodes of pain exposure (18). Most studies examined heel stick or injections as the source of noxious input (4, 17, 19). Skin-to-skin contact is often combined with other methods such as breastfeeding (20, 21) for an even better effect.

There is also some evidence that continuous or longer periods of SSC may diminish chronic or prolonged pain in infants. Prolonged SSC has been associated with a reduction in chronic pain scores measured using the EDIN pain scale and alteration in neuroendocrine markers suggestive of reduced pain (22). Most notably a decrease in dopamine and cortisol levels, and increase in beta-endorphin and serotonin in salivary and urinary samples. An advantage of promoting prolonged periods of SSC is that the parent and infant are already in position to use this pain-relieving method when a painful procedure is required, reducing the need to transfer the infant immediately prior to the painful event.

#### Breastfeeding and breast milk

Thirty-six studies examining the efficacy of breastfeeding for procedural pain relief in infants were included in a recent Cochrane review, most evaluated heel stick, intramuscular injection, and venipuncture. Breastfeeding reduced pain indicators such as duration of crying, heart rate increase and the pain scales NIPS, NFCS and DAN (23). As described above, the pain-relieving effect of breastfeeding can be enhanced when combined with skin-to-skin contact but also with maternal holding (21, 24). Giving the infant expressed breastmilk in isolation of the mother however does not appear to effectively reduce pain scores (23, 25), nor does waiting 1–2 h after the breastfeeding before performing the painful procedure (26).

The pain-relieving effect of breastfeeding persists beyond the neonatal period, at least for immunizations up to 12 months age, with a reduction of bio-behavioural pain responses (27).

#### Parental infant-directed singing and speech

More than a dozen reviews have been published on music-based interventions in neonatal care (28–30), including meta-analyses demonstrating significant positive effects on infant physiological signs, oral feeding volume, stress levels, and maternal anxiety (31, 32). In procedural pain management, previous research with recorded music as well as live lullaby singing carried out by a music therapist, have shown pain relieving and stress reducing effects (33) with a reduction in NIPS pain score (34).

A recent area of research is live or recorded parental infant-directed singing and speech as procedural support (35–43). Infant-directed speech is less effective in reducing distress than infant-directed singing. Parents should therefore sing live to tailor the singing to the infant's state and responses. Parents should sing or hum with a soothing, soft, steady, slow, and constant voice.

Recorded sound of the mother's heartbeats (44–46) or intrauterine sounds (47, 48) may have some effect in reducing physiological and behavioral signs of pain.

More research is needed to confirm the effectiveness of live parental singing and recorded maternal related sounds on infant pain. If used, parents should combine these strategies with other parent-led methods such as SSC and breastfeeding (49).

#### Positioning and touching

Helping the infant to a comforting position during painful procedures is something that easily can be performed by the parents. Facilitated tucking means gently holding the infant in a side lying flexed posture, supported by the hands of the parents. It has been demonstrated to reduce composite scores of stress and pain (50, 51). Holding the baby in the arms of the mother alone has not been associated with effective pain relief; however, in combination with oral glucose or breastfeeding it has been demonstrated to reduce pain intensity scores (24, 52).

Other adjuvant interventions that parents can provide, though with mixed findings on pain reduction, are comforting touch in a structured way (38, 53), e.g., with Yakson touch (54, 55), or combined with other interventions, i.e., as sensorial saturation (56, 57) or nesting (55). A systematic review showed that massage can reduce pain scores and cry duration (58). It is important to combine comforting touch with more effective parent-led interventions.

#### Sweet solution and non-nutritive sucking

While not a parent-led intervention, parents can actively participate by offering a pacifier or finger to suck to their infant following administration of a sweet tasting solution. There is moderate certainty of evidence supporting the pain-relieving effect of a small amount of sweet solution that is given in the mouth prior to a painful procedure, as measured with composite pain scores. Sucrose is the most studied substance (59, 51), followed by glucose (60, 61) and comparisons between the effectiveness of the two substances are inconclusive (62). The effectiveness of sweet tasting solutions has been linked with the concentration, i.e., that it is sweet enough (63). Concentrations of sucrose greater than 18% sucrose may be more effective, however, very high concentrations >35% do not seem to provide greater effect (59). Ensuring that the solution is given on the tip (taste receptors) of the tongue enhances the effect. While significant variation in the dosing of sucrose exists, research supports that small amounts as low as 0.1 ml repeated as necessary throughout the procedure to achieve low pain scores are effective (63). The additive effect of non-nutritive sucking has been associated with enhanced pain-relieving effect of sweet solutions (64) but according to a Cochrane-review, the evidence is of low certainty (59). While the combination of sweet tasting solution with skin-to-skin contact did not appear to provide an additive pain reducing effect in preterm infants undergoing heel stick (65), it is uncertain whether the combination may reduce needle related pain scores in full term infants (6).

### Interaction between the parent and the infant

Infants seek contingent, companionable interactions with their parent. An infant has an innate capacity to communicate, and share affects, which develops through the close relationship with the parents. Naturalistic parent-infant moment-to-moment communication comprises multimodal actions, including music-like vocalizations, and are present in a healthy regulatory parent-infant interaction (66). Parents communicate with their infant in a multimodal and multisensory way where the communication takes place through the intentions and affects carried by the music-like qualities of the infant-parent joint vocalization in combination with the joint dance-like gestures of their bodies, facial movements, and touch (67).

#### Mediating factors in parent-led pain management

Most parent-led interventions are multisensorial in nature. Conceptionally, the Neuromatrix Theory, developed by Melzack (68), an extension to his original “gate theory” of pain developed in 1965 with Wall (69) provides an underlying framework to better understand the possible efficacy of non-pharmacologic methods of pain relief. The model outlines the importance of both ascending and descending inputs to the conscious experience of pain and includes additional inputs such as the important contributions of memory and context. Mediating factors, such as context, relationships, competing multisensorial inputs and meaning have been shown to modulate the experience of pain in adults (70–72). Similarly, infants experience the affective dynamics of pain like adults (73–75). When brain regions within the descending pain modulatory system are more functionally connected, infants have a greater ability to regulate their brain activity in response to incoming noxious stimulation (73). It is not yet determined if the descending pain modulatory system operates at birth in a less functionally connected brain, like the preterm infant's immature brain (73). The descending pain modulatory system is fully functional at birth in term born healthy infants, similar to that observed in adults (73). In the context of parent-led pain care, the combination of the parent's, skin, warmth, voice, breathing rhythm, soothing touch, taste, and scent fully match and harmonize with the infants' multisensory, biopsychosocial state of being, which may modulate the affective dynamics of the infant's pain.

Intuitive parental activities, drawn from the naturally occurring patterns of successful parent-infant communication which support the infant to be heard, seen, and nurtured, can be incorporated into the guidelines for procedural pain management to foster the parent-infant relationship. What seems to be most crucial for an infant before, during and after a painful situation as well as for future painful experiences, is the extent to which the parent is emotionally available and stable, capable of noticing and contingently interpreting cues and communications implicit in the infant's behaviour, responding adequately to the infant's distress signals and being able to soothe, regulate and share the infant's states (49).

### How involved do parents want to be?

Parents' opinions about active contribution in pain management and membership in an interprofessional team-based collaboration, are unanimous across research (4, 76). Parents want to and need to actively participate in their infant's pain management (4). They want to, because parents have a need to feel validated in their role as their infant's principal protector and it helps them feel in control over the painful situation (76). And they need to, because it relieves the parents' own stress when fulfilling their role (51, 76), resulting in an upward spiral towards emotional availability and responsiveness in the painful context. One reason for the effectiveness of parent-led pain management is something as fundamental as love, a parent's love and empathy (77). Parental presence enables a range of effective parent-led pain reducing interventions such as skin-to-skin contact, breastfeeding, rocking and soothing vocalizations.

#### Competence and confidence to participate

Parental advocacy addresses parents' perspectives on advocating for themselves to participate in pain management or advocating for their infant to have their pain managed (78). Advocating for their infant's needs, being involved in shared decision making and having opportunities to provide parent-led non-pharmacological measures, empowers parents to take a more active role in the prevention, assessment and management of their infant's pain and discomfort (79). Consequently, prerequisites for parent-led pain management and equal partnership in the infant's pain care, are parental presence in the NICU and proximity to the infant as well as a trusting and updated staff, and access to information and guidance (5, 78–86).

Parents feel they have a vital role in infant pain care, and they want as much involvement as possible. Both mothers and their partners express a strong desire to be present and involved in various painful procedures to comfort their infant (78, 87). Nevertheless, even though a majority of health care professionals [in some estimates, as many as 70% (88),] find it beneficial for the infant with parents present during common painful procedures i.e., nasogastric tube placement, capillary blood sampling, or venepuncture, the parent's role as a bystander increases the more invasive the procedures are (central line insertion, extubation, lumbar puncture, and intubation) (88–90).

### Challenges of parent-led pain care

While neonatal pain care is moving in the right direction, the culture of care that facilitates active parental contribution in infants' pain management is challenged by structural, organizational, and intra- and interpersonal barriers (85) (Table 1).

**Table 1:** Factors defined in the literature that impede implementation of parent-led pain care (5, 76, 80, 85, 88, 91–94).

Intra- and interpersonal barriers
• Health care professional's attitudes and concerns that parental presence during a procedure increases staff anxiety • Role confusion and power balance in relation to parents' participation • Lack of unified approach among the staff in relieving pain and in parental inclusion • Poor interprofessional collaboration
Organizational barriers
• Lack of policies and protocols which promote parent-led pain relief • Lack of prioritization from the organisation regarding neonatal pain management • Insufficient knowledge and lack of adequate training for health care professionals in effective non-pharmacological methods • Lack of perceived time due to high workload
Structural barriers
• Inadequate paid parental leave policies • Parents’ financial burden of having an infant in neonatal care • Limited physical space within the NICU including lack of family-rooms

Geographical location of the NICU/hospital as well as the health care professional's age, gender, experience, status, education level, expertise, and confidence, can contribute or hamper parent-led pain management (88, 89, 95). In general, nurses appear to be more in favour of involving the parents in pain management compared with physicians and a north-to-south gradient has been observed in Europe, where barriers to the presence of parents and other family members in European NICUs remain higher in southern European countries (88). Lack of communication and collaboration between physicians and nurses is a significant factor in poor pain management (5).

### Enablers of parent-led pain care

Knowledge of providing pain management strategies for their infants empowers parents to take an active role in their infant's pain care, and updated knowledge on parent-led pain-reducing methods is a prerequisite for health care professionals' attitudes towards actively involving parents (76, 80–82, 85, 86, 88–90, 96, 97). Several factors across structural, organizational, and intra- and interpersonal levels have been reported. Research lists numerous factors that can facilitate practice change (Table 2).

**Table 2:** Factors defined in the literature that facilitate implementation of parent-led pain care (5, 81, 86, 91–93, 95, 96, 98–101).

Intra- and interpersonal facilitators
• Well-informed and empowered parents • Professionals' communication skills • Interprofessional healthcare teams which also include parents as active members • Interprofessional collaboration • Professional-parent partnership
Organizational facilitators
• Pain management guidelines and protocols which include parent-led pain reducing methods, parental pain assessment policies, and a plan for parental education • Reflective and progressive management and leadership • Interprofessional education programs • Local pain champions • Collaborative practice
Structural facilitators
• Improved paid parental leave policies • Policymakers, insurers, and hospital systems should financially support parents to access the hospital and their hospitalized infant (housing, transport, parking, childcare) • Family-centred NICU environment with family friendly facilities

### Knowledge transfer

Family-centered care is well recognized for its positive influence on family health outcomes, and there has been advocacy towards parents being considered as essential member of the neonatal care team (102). Despite this trend, the concept of fully integrating families in the provision of infant pain care is a relatively new concept, with poor practice uptake. Many parents remain unaware of their capacity to provide pain relief for their infants which contributes to poor practice (4, 103–106). Nurses and physicians who are key figures in driving clinical change, require education and training that support parental participation (95). The development of professional knowledge consists of developing guidelines, continuous presentation of consolidated new research findings, regular evaluation of pain management outcomes, and periodic education for new staff (91). Knowledge sharing is key for clinical change which leads to successful involvement of parents in their infant's pain management and better pain care for the infants.

#### Parent-targeted education in the neonatal period

Parental education increases parents' knowledge about strategies, self-efficacy and confidence in managing infant pain, and satisfaction with the pain management, and should therefore be incorporated into postnatal care (87). From the parents' point of view, it's a question of timing, framing and content of the knowledge sharing and collaboration. Parents state that they prefer information about pain and pain management to be provided by the staff at a pace directed by the parent and infant, in a calm, supportive and more private environment and without having to ask for it (76, 80). Parents have difficulty absorbing new information during a procedure or in a noisy room with many distractions, and parents might feel hesitant to ask questions or get in the way (76). The initial days of hospitalization are often stressful for NICU parents, but parents express that during the first week of hospitalization it would be optimal for the staff to deliver information on parent-led pain management (78).

Information to increase parent-led strategies needs to be delivered in different formats and in the many languages of our multi-cultural society (83). Research has investigated various knowledge-translational interventions e.g., available on-line videos, educational booklets, pamphlets, dedicated websites, QR codes, educational bundles, customized individual workshop sessions for parents with hands-on guidance in parent-led methods, face-to-face interaction with health care professionals (78, 83, 84, 87, 107–109). Parents (especially low literacy parents) may need better visual aids to fully comprehend the various comfort techniques (107). Even though many parents prefer electronic resources, parents still desire human interaction for learning and favor a health care professional to review the information with them face to face (78).

Co-creation of mutual knowledge is a dynamic process that is important for the establishment of a nurse-parent partnership (110) which increases the sense of equality among parents and helps them feel valued, trusted, respected, and seen as a resource (86). Open and honest communication is the basis for cooperation in pain management (110), including health care professionals' willingness to listen to the parents to find a mutually satisfactory course of action (86). Parents want for example guidance on how to interpret an infant's pain and guidance in pain assessment. A crucial factor is the freedom for the parents to choose and being listened to about how much, and in which way, they want to participate—or not (86).

#### Online e-health resources for parents

In addition to formal (e.g., healthcare providers) and informal (e.g., peer groups) written and oral resources to advise parents of the importance of their role, there has been a trend towards using online e-health resources to inform and engage parents in pain care. Parents in neonatal care have reported high use and a preference for using the internet or smartphone to access information (111, 112). However, there remains a paucity of quality online resources. An evaluation of websites or mHealth apps targeted to parents of preterm infants found issues related to their credibility and moderate overall quality, with less than 11% providing any information on infant pain (113, 114). While online resources appear to be favoured by parents, parents recognize possible issues regarding the quality of the content. Parents reported that online resources created by institutions with healthcare provider input would reduce this concern.

Additionally, only 11 studies were included in a comprehensive analysis of the impact of parent-targeted eHealth educational interventions about infant procedural pain management on parental (e.g., mental health, knowledge uptake), eHealth (e.g., acceptance, use), and pain management outcomes [e.g., parental involvement, infant pain response (115)]. Findings revealed that short and engaging educational videos may positively impact parents' knowledge, confidence, and desire to be involved in procedural pain management for their infant (109, 116). Key facilitators of e-health related resources include alignment with the Baby-Friendly Hospital Initiative, modeling by Lactation Consultants, and frequent reminders (117). However, there remains a gap in our understanding if the provision of this information to parents leads to a greater parental involvement in clinical practice and subsequent reduction on infant pain. Further work in this area is warranted.

### Practice and policy implications

To date, few studies in the neonatal pain context, have investigated parents' experiences of providing parent-led pain management for their infant (4). However, several studies are ongoing. The study “Parents as pain management in Swedish neonatal care (SWEpap)” (118) is a cutting-edge interdisciplinary multi-centre clinical study with mixed methods involving both parents and health professionals. The aim of SWEpap is to investigate parents' and nurses' reflections on experiencing parent-delivered pain management, breastfeeding (when applicable), skin-to-skin contact, and parental infant-directed lullaby singing, during painful procedures in neonatal care. Another on-going study, the British Petal Trial, is examining the pain-relieving effects of parental touching, or more specific strokes in 112 neonates undergoing heel lancing on noxious-evoked brain activity, pain scores, parental anxiety, and parents' reflections on participating in neonatal pain research (119, 120).

In many countries parents have limited possibilities to stay with their newborn infant at hospital, due to social support systems, limited space at the unit or rules and tradition. Therefore, some researchers are trying to develop systems with recorded maternal voices (121, 122) or robots (123, 124) to replace the mother when not there. This “better-than-nothing” approach could however delay efforts to include parents in the hospital care of their infants, especially when resource-saving arguments are being used (125).

#### Recommendation for guidelines

There has been a considerable movement towards ensuring that parents are included in pain standards and guidelines. The Pediatric Pain Management Standard released in April 2023 by the Health Standards Organization of Canada in partnership with Solutions for Kids in Pain, a government funded national centre for excellence is aimed to support the delivery of equitable evidence informed pain care. The standard consisting of 34 recommendations recognizes that parents are equal members of the team and should be actively involved in decision making and care decisions (126). The standard is well aligned with the Lancet commission call to action to improve pediatric pain care (127). Despite these important inroads, few guidelines issued by a national or international authority, or a professional organization or network specifically recommend or include implementation strategies for parent-led pain management, with even fewer addressing parent-led interventions in low income and low-tech settings (4).

When written instructions are available in the NICUs about pain assessment with a structure for pain documentation, and the use of non-pharmacological methods including parent-led interventions, the staff reports higher awareness on these matters than units that do not have written local pain management guidelines for everyday work (93). One of the main obstacles leading to inadequate use of non-pharmacological methods is the lack of standards and guidelines. Guidelines are important means to improve the effectiveness and consistency of care. However, the mere existence of guidelines does not guarantee the use of them (128). Adherence depends on for example ongoing training and monitoring of their use (90). Regular rotations of staff means that frequency of teaching needs must be evaluated and prioritized to increase awareness of parent involvement in pain care, pain assessment, assessment tools and parent-led interventions.

Guidelines are usually initiated and implemented in health care through a top-down approach where information filters down through a hierarchical structure. When this approach is combined with dedicated and well-informed nurses and physicians as local pain champions in each NICU, a profound long-lasting change can happen. This will improve the conditions for the pain exposed infant and welcome the parents as partners in neonatal pain management. Local pain facilitators strive to provide health care professionals the opportunity to talk about and reflect on pain management issues and professional limitations and needs (91). Interprofessional discussions will build confidence, maintain skills, and produce living guidelines. One method to support the uptake of practice recommendations, is through quality improvement initiatives, a strategy that has been emphasized in the Canadian Pediatric Standards (126).

## Conclusion

Parent-led neonatal pain management is more than simply a humane and compassionate thing to do. The inclusion of parent-led pain care has been scientifically proven to be one of the most effective ways to reduce pain associated with repeated painful procedures in early life and parents report a desire to participate. Focus on enablers across inta- and interprofessional, organizational and structural levels and implementation of recommended pediatric pain guidelines can support the provision of optimal evidence-based family-centered neonatal pain management.
